# Prenatal care utilization for fetuses with spina bifida in California

**DOI:** 10.1186/s12884-025-07800-z

**Published:** 2025-07-03

**Authors:** Than S. Kyaw, Natalia Leva, Debbie Goldberg, Isabel Elaine Allen, Lindsay A. Hampson, Hillary L. Copp

**Affiliations:** 1 Medical Scientist Training Program, University of California San Francisco, San Francisco, CA USA; 2 Urology Department, San Francisco Medical Center, Kaiser Permanente, San Francisco, CA USA; 3 Department of Epidemiology & Biostatistics, University of California San Francisco, San Francisco, CA USA; 4 Department of Urology, University of California San Francisco, San Francisco, CA USA

**Keywords:** Prenatal care, APNCU index, Spina bifida, Neural tube defects

## Abstract

**Background:**

To (1) compare prenatal care (PNC) utilization of women carrying fetuses with and without spina bifida (SB), (2) identify factors associated with less than adequate PNC in the SB group, and (3) correlate neonatal complications with PNC utilization in the SB group.

**Methods:**

Retrospective cohort study using data from the California Department of Health Care Access and Information Database for all liveborn infants in 2005–2012. We compared women carrying fetuses with SB to women carrying fetuses without SB. The primary outcome was PNC utilization, assessed using the Adequacy of Prenatal Care Utilization (APNCU) Index. Univariate and multivariable analyses identified factors associated with less than adequate PNC. Associations between neonatal morbidity and PNC utilization were examined.

**Results:**

Among 1,049 SB birth records and 4,045,262 non-SB birth records evaluated, intensive PNC utilization was higher in women carrying SB fetuses compared to those without SB fetuses (47% vs. 37%, *p* < 0.0001). However, more than half (53%) of women with SB fetuses did not receive intensive PNC and 21% received less-than-adequate care. Both univariate and multivariate analyses of women with SB fetuses showed that having non-private insurance was associated with less than adequate care (OR 2.3; 95% CI: 1.6,3.2; *p* < 0.01). Intensive PNC was linked to higher rates of neonatal complications, including prematurity and low birth weight (69% vs. 6–14%; *p* < 0.001) in the SB group.

**Conclusions:**

Although a substantial number of women with SB fetuses received intensive PNC utilization, over half did not. Despite recommendations for close monitoring, 1 in 5 women with SB fetuses did not receive adequate care, which was associated with having non-private insurance. This identifies a modifiable target to improve care and suggests the need for further studies to examine this association.

## Background

Spina bifida (SB) is a prevalent congenital malformation caused by improper closure of the neural tube early in embryonic development, which can be detected in routine prenatal screening tests [1]. It is often associated with paralysis, sensory deficits, orthopedic abnormalities, and bowel and bladder dysfunction [1]. Since this is a diagnosis that requires lifelong medical care, there is great interest in understanding how to improve care for patients with SB, including care that begins prenatally for mothers with a SB fetus. Preventative strategies such as folic acid supplementation prior to conception in 1996 have reduced the risk of neural tube defects from 5 to 10 per 10,000 live births to 2-2.6 per 10,000 live births [2, 3].

Fetuses with congenital anomalies, including SB, have a significantly increased risk of stillbirth, with the risk for SB fetuses being 4 times higher than for the general pregnancy population [4]. To aid in comprehensive counseling and management of SB pregnancies, the American College of Obstetrics and Gynecology (ACOG) and the Spina Bifida Association published Clinical Management Guidelines and Prenatal Counseling Guidelines [5–7]. When SB is suspected, patients should be referred to high-risk obstetricians in the maternal-fetal medicine unit for individualized care and further management [7]. Some academic institutions recommend monthly growth scans and weekly non-stress tests if the fetus has a normal growth trajectory or twice weekly if the fetus is below the 10th percentile for overall weight [8]. Despite these guidelines emphasizing attentive monitoring, the extent of prenatal care (PNC) received to manage such a complex, high-risk pregnancy remains unclear.

This study aims to understand (1) the level of PNC utilization in mothers with a SB fetus compared with mothers without a SB fetus, (2) factors contributing to poor PNC utilization in the SB group, and (3) the neonatal outcomes for mothers with a SB fetus. By utilizing the Adequacy of Prenatal Care Utilization (APNCU) index, which defines levels of care based on the timing of initiation and frequency of PNC [9], we hypothesized that the majority of women carrying fetuses with SB are receiving intensive PNC. Herein, we seek to characterize PNC utilization and understand factors associated with less than adequate PNC utilization among women with SB fetuses.

## Methods

### Study design

We performed a retrospective cohort study to examine the level of PNC utilization among women carrying fetuses with SB compared with women carrying fetuses without SB in California using Linked Birth Files data from the California Department of Health Care Access and Information (HCAI) database. The comparison control group consists of all non-SB fetuses. From 2005 to 2012, HCAI created Linked Birth Files to study delivery and birth outcomes in California by linking maternal and neonatal records [10]. This database includes records from hospitals that had comprehensive maternal and neonatal data, linking hospital records of mothers and infants to provide insight into birth outcomes and healthcare utilization in California.

**Fig. 1:**
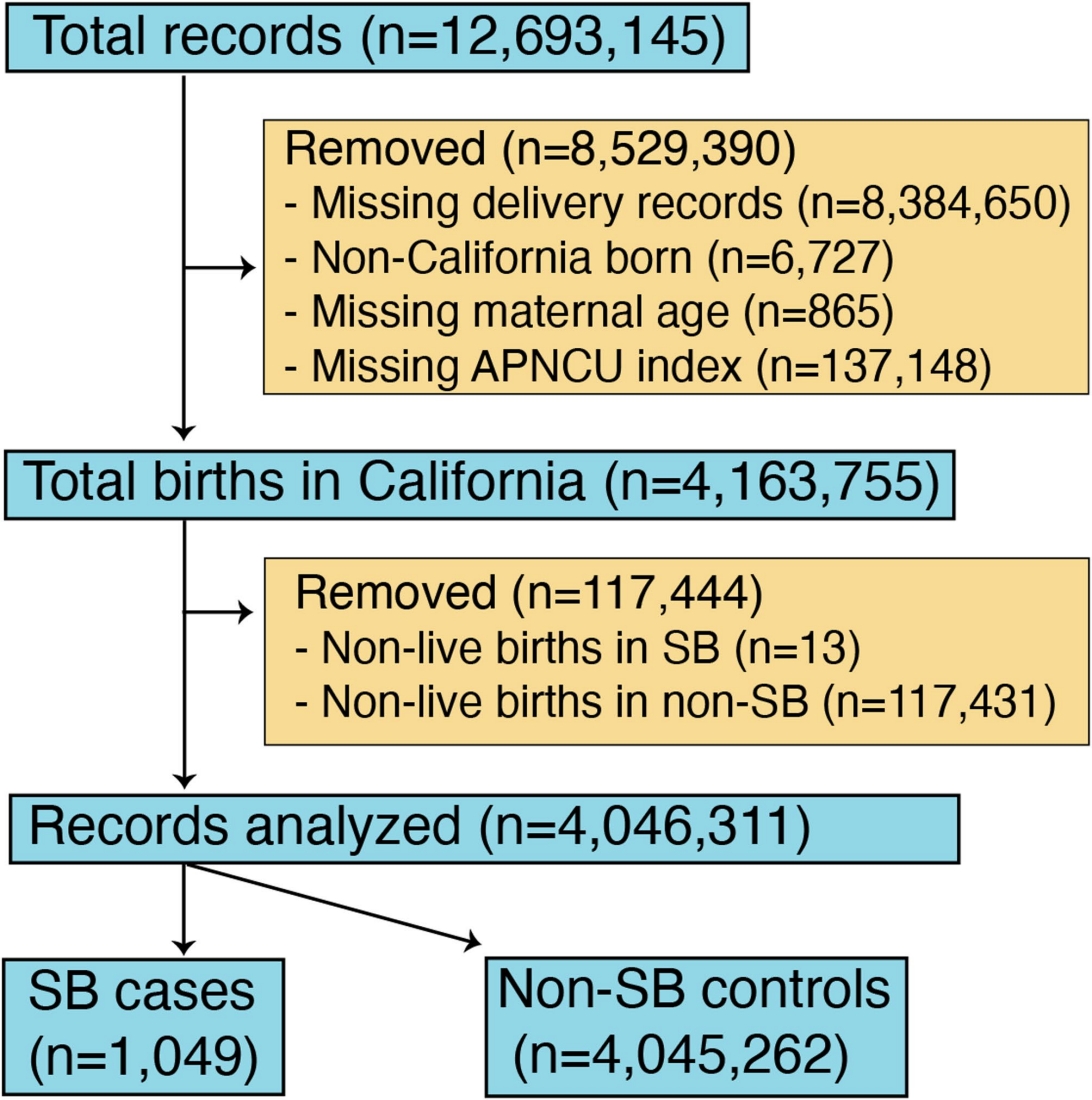
Derivation of our study population obtained from the California Department of Health Care Access and Information database of Linked Birth Files. APNCU stands for Adequacy of Prenatal Care Utilization Index

Using the Linked Birth Files from the HCAI database, we restricted our analysis to all women with liveborn infants in California between January 1, 2005, and December 31, 2012 (Fig. 1). Births outside of California (0.5% of total records), non-live births including stillbirths, miscarriages, and terminated pregnancies (0.9% of total records), and cases with insufficient data to calculate the APNCU index (67% of total records) were excluded (Fig. 1). Births outside California were excluded due to a disproportionately large amount of missing information in these cases. Non-live birth is a potential confounder as it eliminates the need for further PNC upon occurrence. The International Classification of Diseases, 9th revision, Clinical Modification codes [11] were used to define liveborn infants (codes V30-V39) and SB (codes 740.01-741.03, 741.90-741.93, 756.17).

Our study was approved by the California Committee for the Protection of Human Subjects Institutional Review Board (CPHS IRB #00000681) and complies with the World Medical Association’s Helsinki Declaration. De-identified individual-level data were stored in encrypted, secure storage designed for sensitive medical data and accessed only by individuals trained in HIPAA compliance to ensure patient confidentiality.

### Outcomes of interest

#### Primary outcome (PNC utilization)

##### *Prenatal care* (PNC) utilization

The primary outcome variable investigated was the level of PNC. The level of PNC utilization was defined using the Adequacy of Prenatal Care Utilization (APNCU) Index which captures the timing of PNC initiation and frequency of visits received after initiation [12]. ACOG recommends one visit every month for the first 28 weeks of gestation, a visit every 2 weeks through 36 weeks, and weekly thereafter until delivery for uncomplicated pregnancies [12]. When SB is diagnosed in the second trimester between 15 and 20 weeks of gestation, ACOG recommends referrals to maternal-fetal medicine specialists and tertiary neonatal care centers for additional tests and management [7]. Antenatal fetal surveillance and serial ultrasound examinations may be considered for monitoring fetal growth and hydrocephalus [7].

**Fig. 2:**
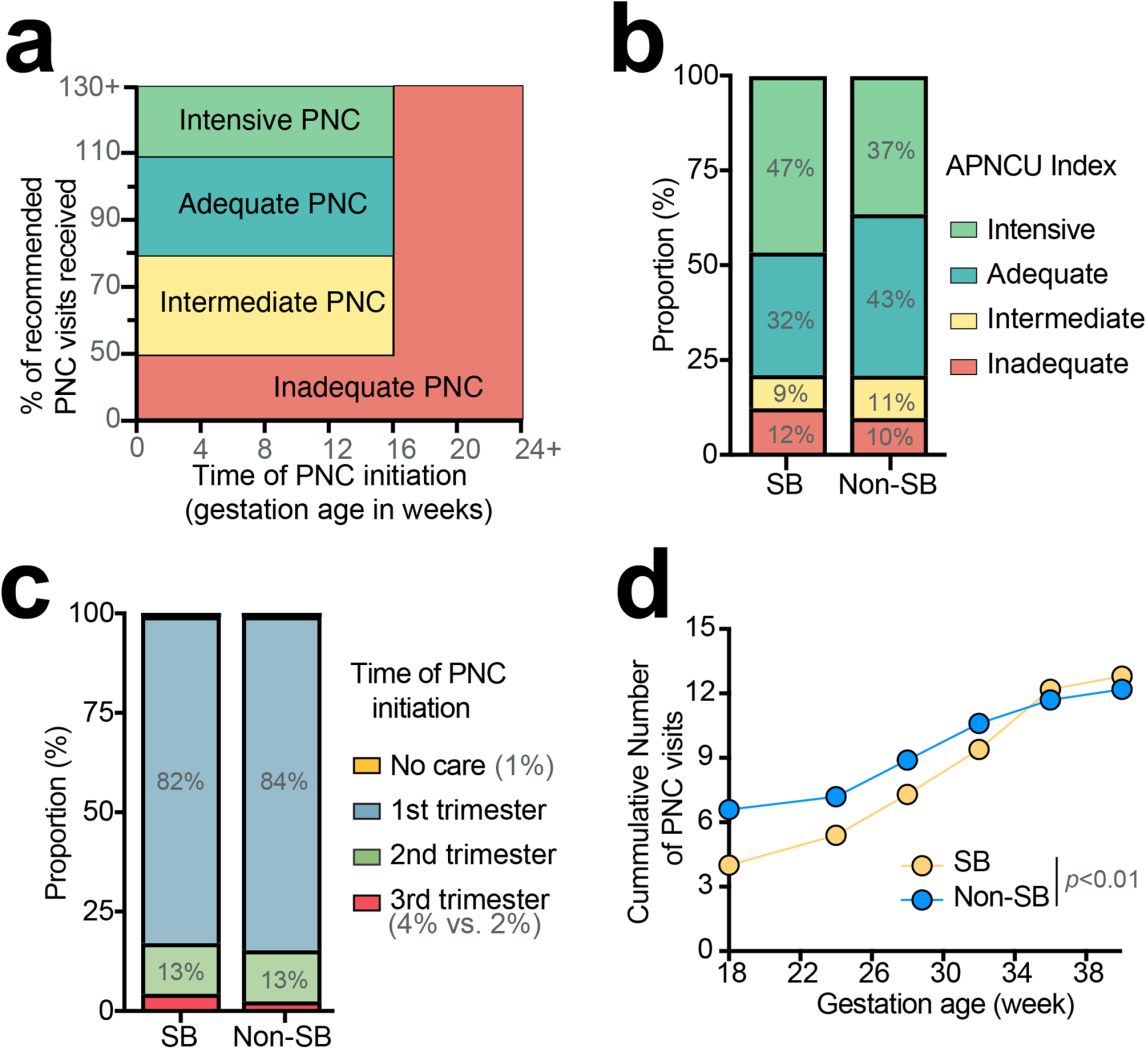
Adequacy of prenatal care (PNC) in Spina Bifida (SB) births in California between 2005–2012. (**a**) Categories of PNC utilization based on the Adequacy of Prenatal Care Utilization (APNCU) index, which considers the time of PNC initiation and frequency of PNC visits. The expected frequency of PNC visits is adjusted based on gestation age at birth. (**b**) Proportion of PNC utilization based on APNCU index. (**c**) Time of PNC initiation. (**d**) Rate of PNC utilization across gestation age

The APNCU index divides PNC utilization into 4 groups: inadequate, intermediate, adequate, and intensive (adequate plus) [12, 13] (Fig. 2a). Inadequate PNC is defined as starting PNC after the 4th month of pregnancy or receiving less than 50% of recommended visits [12]. Intermediate care means PNC initiation by the 4th month of pregnancy with 50–79% of visits completed [12]. Adequate PNC begins by the 4th month of pregnancy with 80–109% of expected visits completed [12]. Intensive PNC requires initiation of care between 1 and 4 months of pregnancy, with ≥ 110% expected visits received [12]. The recommended number of PNC visits was calibrated to the gestation age at birth; 12 visits were expected for full-termed infants born at 38 weeks and fewer visits for pre-termed infants born < 37 weeks.

Key variables, such as “Number of prenatal care visits”, “Month prenatal care visits began”, and “gestational age in weeks” from the HCAI database were used to calculate unadjusted expected prenatal care visits, expected prenatal care visits adjusted based on gestational age at birth, expected visit ratio, expected visit index, and month prenatal care initiation index. These calculations were then categorized into APNCU Index.

#### Secondary outcomes

##### Neonatal morbidity

Neonatal morbidity was assessed as a secondary outcome. Neonatal morbidity was defined using the metric “Unexpected Complications in Term Newborns”, developed by the California Maternal Quality Care Collaborative [14]. Although this metric was originally developed for term newborns, its list of adverse neonatal conditions associated with labor and delivery management is also applicable towards preterm and high-risk infants [15]. Prematurity (< 37 weeks of gestation) and low infant birth weight (≤ 2500 g) were included as indicators of poor neonatal outcomes [16].

#### Predictor variables

##### Demographics

Maternal age was categorized as < 20 years (very young mothers), 20–34 years (referent group), and ≥ 35 years (mothers with advanced age [17]) based on previous research identifying unique risk factors within each group to assess PNC utilization [18]. Race was categorized as White, Black, Asian/Pacific Islander, Multiracial, and Other/Unknown. Ethnicity (Hispanic/Non-Hispanic) was not available. Mother’s birthplace was categorized as California, United States (outside California), Mexico, and foreign-born. Mother’s insurance was categorized as private (health maintenance organization and preferred provider organization coverage) and non-private (government forms of coverage and self-pay).

##### Maternal health factors

Maternal pre-pregnancy body-mass-index (BMI) was categorized as underweight (< 18.5), normal (18.6–24.9), overweight (25–29.9), and obese (30–55.9), and outliers (56+). Maternal pre-pregnancy diabetes was also recorded. The mode of delivery was dichotomized into vaginal and cesarean.

##### Socioeconomic status (SES)

Neighborhood scores were created at the level of the zip code tabulation area using United States Census data [19, 20]. We measured SES by assigning a neighborhood score to each patient using the Diez-Roux method [21].

### Statistical analysis

Descriptive analyses were performed to compare the characteristics of women carrying fetuses with and without SB. Chi-squared tests and 2-tailed Student’s independent group t-tests were used to compare the percentages and means, respectively. Continuous variables were reported as means and standard deviations, whereas categorical variables were reported as frequencies and percentages. Multivariable logistic regression models were used to identify factors associated with less than adequate PNC (defined as inadequate or intermediate PNC utilization). Based on the associations found in the literature, maternal age, race, and insurance status were selected a priori for inclusion in the final multivariate model. Maternal age at delivery, mode of delivery, parity, maternal birthplace, insurance status, and Diez-Roux scores were assessed for inclusion in the final multivariate model based on the significance of their univariate analysis. Chi-squared tests were used to compare the neonatal outcomes between the SB and non-SB groups, stratified by PNC utilization. All analyses were performed with SAS, version 9.4.

## Results

### Study population

Among 12,693,145 records in California from 2005 to 2012, there were 4,163,755 birth records in California with sufficient data to calculate the APNCU index. Of these, 13 non-live births were identified in the SB group (1.2% of total SB births) and 117,431 non-live births in the non-SB control group (2.8% of total non-SB births; Fig. 1). These non-live births were excluded as they eliminated the need for PNC upon discovery. After exclusions, the final analysis included 4,046,311 records, comprising 1,049 SB births and 4,045,262 non-SB births (Fig. 1). The majority of mothers with SB fetuses were aged 20–34 years (73%), White (82%), and non-smokers (71%). Most infants with SB were born via cesarean delivery (65%), at term (78%), with normal birth weight (77%) (Table 1).

**Table 1:** Patient characteristics among spina bifida (SB) and non-SB births from 2005–2012

Characteristics	All births		Non-SB controls		SB births	
	Total (%)	*p*-value*	*N*	%	*N*	%
Number of births	100		4,045,262	100	1049	100
**Maternal factors**						
**Maternal age at delivery (yr)**		0.608				
< 20	9		355,454	9	101	10
20–34	73		2,959,284	73	769	73
≥ 35	18		730,524	18	179	17
**Mother’s race**		< 0.001				
White	76		3,064,724	76	862	82
Black	5		214,916	5	41	4
Asian/Pacific Islander	13		510,692	13	63	6
Multiracial	3		104,669	3	27	3
Other	2		85,397	2	27	3
Unknown	2		64,864	2	29	3
**Maternal birthplace**		< 0.001				
California	46		1,856,079	46	469	45
US, not CA	10		401,617	10	97	9
Foreign born	44		1,783,599	44	483	46
Unknown	< 1		3,967	< 1	0	0
**Mother’s pre-pregnancy Body-mass-index (kg/m**^**2**^)		< 0.001				
16.0-18.5	3		121,187	3	11	1
18.6–24.9	33		1,351,934	33	284	27
25.0-29.9	18		711,657	18	195	19
30.0-55.9	14		566,715	14	199	19
Outlier/Unknown	32		1,293,769	32	360	34
**Maternal diabetes**		0.043				
Yes	1		31,709	1	14	1
No	99		4,013,553	99	1,035	99
**Socioeconomic status**						
**Mother’s insurance**		< 0.001				
Private	49		1,964,673	49	394	38
Non-Private	51		2,058,357	51	646	62
Unknown/Missing	< 1		22,232	< 1	9	< 1
**Neighborhood SES (Diez Roux)**		< 0.001				
Lowest 10% (lower SES)	10		406,492	10	151	14
Highest 90% (higher SES)	87		3,498,766	87	836	80
Unknown	3		140,004	3	62	6
**Delivery outcome**						
**Delivery mode**		< 0.01				
Vaginal	67		2,725,958	67	362	35
Cesarean	33		1,319,303	33	686	65
Missing	< 1		1	< 1	1	< 1
**Prematurity**		< 0.01				
Extreme prematurity (< 28.0 weeks)	1		23,222	1	20	2
Premature (28.0-36.9 weeks)	10		415,247	10	211	20
Term (≥ 37.0 weeks)	89		3,606,407	89	817	78
Unknown	< 1		386	< 1	1	< 1
**Birth weight (grams)**		< 0.01				
400–1499	1		43,313	1	37	4
1500–2499	6		226,219	6	140	13
2500–3999	85		3,435,925	85	807	77
4000–6000	8		337,593	8	60	6
Outlier/Unknown	< 1		2,212	< 1	5	< 1
**Sex of infant**		0.16				
Male	51		2,070,843	51	506	48
Female	49		1,974,390	49	543	52
Unknown	< 1		29	< 1	0	0

### PNC utilization in SB cases and non-SB controls

Using the APNCU index to categorize PNC utilization (Fig. 2a), a significantly higher number of women carrying fetuses with SB had intensive care (47% vs. 37%; *p* < 0.01; Fig. 2b) as we would expect to see in accordance with recommended care guidelines. However, a disproportionally higher number of SB cases also had inadequate care compared to non-SB controls, highlighting heterogeneities in PNC utilization within the SB group (12% vs. 10%; *p* < 0.01; Fig. 2b).

The pattern of PNC utilization also differs between the SB group and the non-SB control group. SB group had significantly fewer PNC visits earlier in the pregnancy compared to the non-SB controls (Fig. 2d). A disproportionately higher number of women carrying fetuses with SB initiated PNC in the third trimester (4% vs. 2%; *p* = 0.001; Fig. 2c). However, as the pregnancy progressed, the rate of PNC utilization significantly increased (*p* < 0.01; Fig. 2d). The utilization rate then plateaued around 36 weeks of gestation (Fig. 2d).

### Factors associated with less than adequate PNC in SB cases

**Fig. 3:**
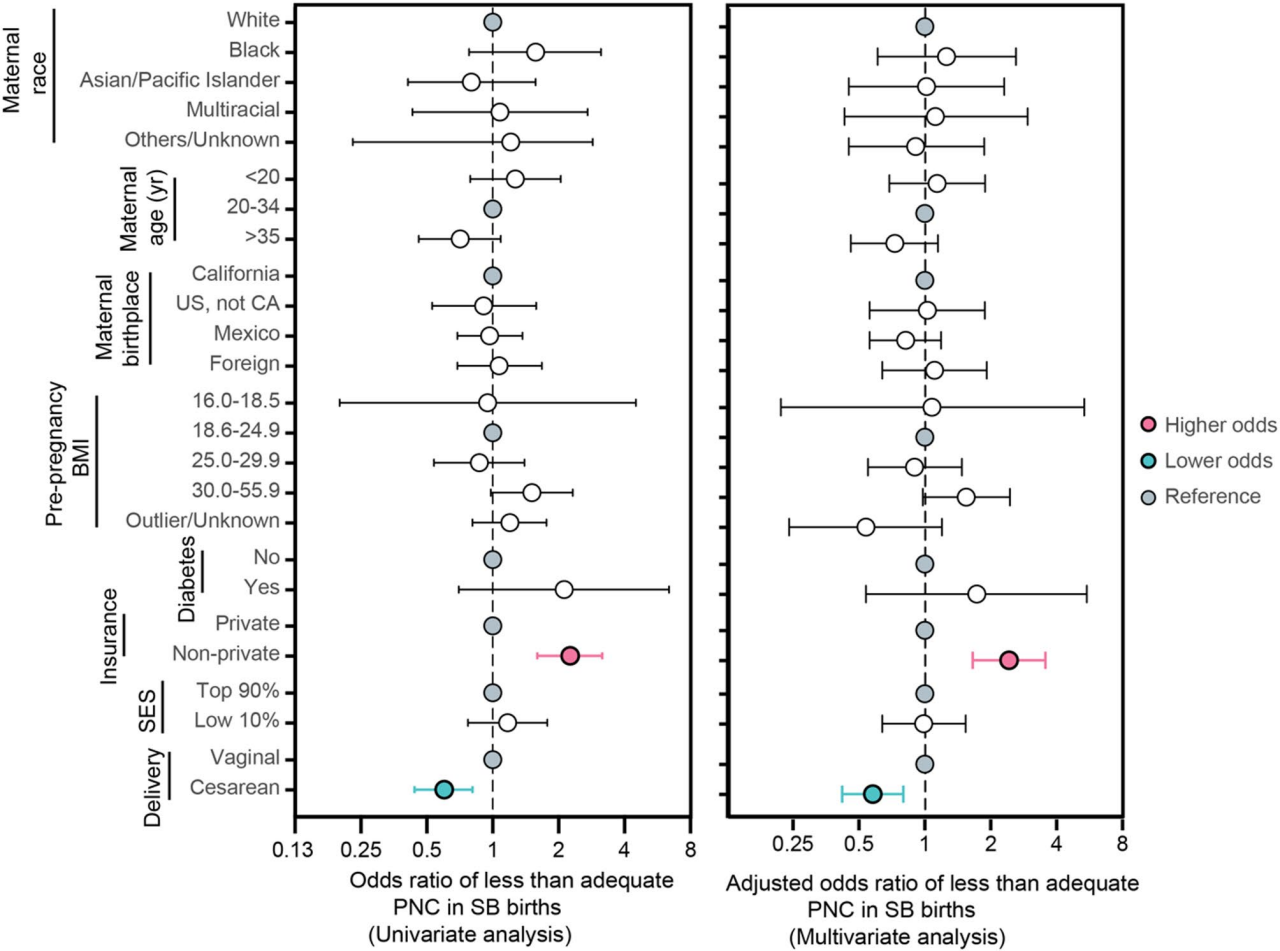
Univariate (left) and multivariate (right) analysis of risk factors associated with insufficient prenatal care (PNC) in Spina Bifida (SB) births in California between 2005–2012. BMI means body mass index measured in kg/m^2^. Neighborhood socioeconomic status (SES) was calculated using Diez-Roux metrics. Multivariate analysis adjusted for maternal race, maternal age, maternal insurance status, neighborhood SES, and mode of delivery

Although 47% of women carrying fetuses with SB received the recommended intensive care, 21% did not receive adequate care (Fig. 2b). To understand the driving force behind such differences in PNC utilization, we examined sociodemographic factors associated with less-than-adequate PNC utilization in the SB group. Among many factors analyzed, non-private insurance was a strong predictor of less-than-adequate PNC in SB cases (adjusted OR 2.3; 95 CI1.6-3.2; Fig. 3) and remained significant after adjusting for maternal age, mode of delivery, parity, maternal birthplace, insurance status, and Diez-Roux scores. Notably, 75% of SB cases with less-than-adequate PNC had non-private insurance (Fig. 3). Cesarean delivery was associated with lower odds of obtaining less-than-adequate PNC compared to vaginal delivery on adjusted analysis (adjusted OR 0.6; 0.4,0.8; Fig. 3).

### Neonatal outcomes associated with PNC utilization in the SB cases

Overall, 22% of all babies born with SB developed neonatal complications (Table 2). Comparing the rates of complications in different APNCU groups, the group with the highest PNC utilization (intensive) had a higher rate of complications compared to other groups (69% in intensive vs. 6–14% in all others; *p* < 0.001; Table 2). The rate of prematurity and low birth weights in SB births were also significantly higher in the intensive group (*p* < 0.001, Table 2).

**Table 2:** Neonatal morbidity in spina bifida (SB) births. A list of unexpected complications was developed by the California maternal quality care collaborative. Prematurity is defined as < 37 weeks of gestation and very low birth weight is ≤ 2500 g

	Unexpected complications		Prematurity		Very low birth weights	
	*N*	%	*N*	%	*N*	%
**Adequacy of Prenatal Care Utilization Index**						
Inadequate	32	14	32	14	4	11
Intermediate	14	6	14	6	3	8
Adequate	25	11	24	10	4	11
Intensive	162	69	161	70	26	70

## Discussion

In this study, we compared PNC utilization between women carrying fetuses with and without SB. Given that SB is considered a high-risk pregnancy requiring close monitoring, we identified risk factors associated with less-than-adequate PNC within the SB group. We showed that intensive PNC utilization was significantly higher in women carrying SB fetuses compared to those without SB fetuses. However, more than half (53%) of women with SB fetuses did not receive intensive PNC and 21% received less-than-adequate care with delayed care in the first trimester. These findings are concerning given that close monitoring and intensive care are recommended in this vulnerable, high-risk population of pregnant women. We further found that less-than-adequate care is associated with having non-private insurance among women with SB fetuses. Intensive PNC was linked to higher rates of neonatal complications, including prematurity and low birth weight; this likely reflects the severity of SB and its associated comorbidities.

Despite > 99% having documented insurance coverage and the need for intensive PNC, 1 in 5 pregnant women with SB fetuses did not receive adequate prenatal care. Non-private health insurance was a significant risk factor for less-than-adequate PNC, suggesting that insurance alone does not ensure access to healthcare. Although California provides universal access to PNC through the Medi-Cal Access Program [22], our findings highlight that providing access is not enough to eliminate healthcare inequalities. Different patterns of PNC utilization in women with SB fetuses, specifically delayed care in the early gestation period with increased care in the third trimester, may suggest a lack of access to preconception/family planning and initiation of folic acid supplementation as recommended.

Possible barriers that hinder PNC utilization may include work/family obligations, a lack of reliable transportation, living in areas with a scarcity of services, long wait times, and insurance-based discrimination. One study comparing individuals with private insurance and Medicaid (non-private insurance) showed that Medicaid beneficiaries experienced more barriers to timely primary care [23]. For example, Medicaid beneficiaries were disproportionately affected by the lack of reliable transportation, which led to both delayed and foregone care in low-income populations [24]. Poor experiences with the healthcare system, such as longer wait times, difficulty finding clinics that accept Medicaid, and perceived discrimination based on insurance may further discourage patients from seeking preventive health services [24]. Future studies examining the reasons for less-than-adequate PNC utilization in mothers with SB babies with non-private health insurance will be critical to combat healthcare inequalities.

Our study has limitations. One major limitation is that many records had missing information to calculate the APNCU Index, which is our primary objective. Two-thirds of the total records identified had missing delivery records, which made drawing definitive conclusions challenging. Cases with missing delivery records may have included elective termination cases, which could account for low percent of stillborn observed in our study compared to higher rates reported in meta-analyses [25]. However, it is reassuring that the percentage of missing records is similar in the SB cases and non-SB controls, reducing but not eliminating potential biases. Although we chose to exclude non-live births from our PNC utilization analyses to avoid confounding factors, this decision may introduce biases, as stillbirth may be the ultimate complication of neglected prenatal care. Nevertheless, these cases accounted for < 1% of total records and their inclusion did not significantly change the trends of PNC utilization reported in our study (*p* > 0.05). Additionally, demographic differences between SB and control populations seen in our study may reflect real-world variations in SB prevalence [2, 3]. While a matched cohort analysis could enhance comparability, we accounted for key confounders through multivariable regression, preserving statistical power while maintaining generalizability [26].

Another major limitation is that the HCAI Linked Birth Files did not provide the severity of SB, which limits our ability to assess the associations between disease severity and PNC utilization. It contains census-level data, which means we can only infer an individual’s SES using the Diez Roux scores based on the neighborhood in which patients reside. Although the APNCU index captures the timing and frequency of PNC, it does not account for the content or quality of PNC delivered, which is important in optimizing pregnancy conditions [27].

Despite these limitations, our large, diverse statewide sample provides valuable insights into PNC utilization among women with SB fetuses and emphasizes a specific at-risk population that can be followed in future studies. With the fetal surgical interventions available for the management of myelomeningocele at 18–25 weeks of gestation age [28], it is even more critical that pregnant mothers with SB fetuses receive early and frequent prenatal care to qualify for and optimize fetal outcomes. Future studies will continue to track prenatal care utilization in women with SB fetuses and determine if insurance status remains correlated with delayed prenatal care.

## Conclusions

Our study showed that although approximately half of all women carrying fetuses with SB had guideline-directed intensive care, there was still a significant portion of women who received less-than-adequate care with delayed and less frequent prenatal visits compared to the non-SB controls. Non-private insurance is associated with less-than-adequate prenatal care utilization in women carrying fetuses with SB, which likely underestimates the magnitude of the issue given women with SB fetuses should be receiving intensive care. This highlights the need for targeted interventions that address barriers to ensure equitable care. Future research will explore strategies to overcome such challenges and improve prenatal care for this vulnerable population.

## Data Availability

The datasets generated and/or analyzed during the current study are not publicly available due to licensing restrictions from the California Department of Health Care Access and Information Database (HCAI), but are available from the corresponding author on reasonable request.
